# The impacts of including information about the number of carcinogens in smoke on standardized cigarette packs in the UK

**DOI:** 10.1093/eurpub/ckab101

**Published:** 2021-09-14

**Authors:** Crawford Moodie, Catherine Best, Nathan Critchlow, Sara Hitchman, Martine Stead, Ann McNeill

**Affiliations:** 1 Faculty of Health Sciences and Sport, Institute for Social Marketing and Health, University of Stirling, Stirling, Scotland; 2 National Addiction Centre, Institute of Psychiatry, Psychology and Neuroscience, King’s College London, London, England

## Abstract

**Background:**

Since May 2017, standardized packaging has been mandatory in the UK, with packs required to display an ‘information message’ explaining that there are more than 70 carcinogens in tobacco smoke.

**Methods:**

Three waves of a longitudinal online survey in the UK with smokers pre-standardized packaging (Wave 1: April–May 2016) and followed up post-standardized packaging (Wave 2: September–November 2017, Wave 3: May–July 2019). Of the 6233 smokers at Wave 1, 4293 responded at Wave 2 and 3175 at Wave 3. We explored knowledge of the number of carcinogens in smoke, and whether knowing that smoke contains more than 70 carcinogens mediated change in the belief that the dangers of smoking are exaggerated (risk perception), stubbing out cigarettes, quit intentions and quitting. As the information message is larger on roll-your-own packs than on cigarette packs, as the packs are larger, we also explored whether there was any difference in knowing that smoke contains more than 70 carcinogens between exclusive cigarette smokers and exclusive roll-your-own smokers.

**Results:**

Knowledge that there are over 70 carcinogens in smoke increased among smokers across waves, with the increase from Waves 1 to 3 greater for exclusive roll-your-own smokers than exclusive cigarette smokers (adjusted odds ratio=1.44; 95% CI 1.03–2.03). Knowledge that there are over 70 carcinogens in smoke mediated higher risk perception but not stubbing cigarettes out, quit intentions or quitting.

**Conclusions:**

The information message improved knowledge of how many carcinogens are in smoke, particularly among exclusive roll-your-own smokers, and this was linked to higher risk perception.

## Introduction

Smoking remains a leading cause of death and disability in Europe, with the prevalence of tobacco use higher than in any other World Health Organization (WHO) region.^1^ There has been considerable progress in strengthening tobacco control in Europe,^1^ particularly as a result of the Tobacco Products Directive (TPD).^2^ The TPD has had a significant impact on the appearance of packs of cigarettes and roll-your-own (RYO) tobacco in the European Union (EU). Cigarettes must be sold in cuboid packs or shoulder boxes and contain a minimum of 20, while RYO must be sold in cuboid packs, shoulder boxes, pouches or cylinders and contain a minimum of 30 g. For both products, any reference on packs to taste, smell and flavour is banned, and packs must have pictorial warnings on the primary display areas and a general warning (‘Smoking kills’ or ‘Smoking kills—quit now’) and an information message (‘Tobacco smoke contains over 70 substances known to cause cancer’) on the secondary display areas.^2^ The TPD also permitted countries to go further than the minimum pack requirements and introduce standardized (or plain) packaging, with France (1 January 2017), the UK (20 May 2017), Ireland (20 September 2018), Slovenia (1 January 2020) and Belgium (1 January 2021) having fully implemented this policy. The focus of this article is on the information message on standardized packs in the UK.

Although consumers want more information about the constituents in tobacco smoke,^3^^,^^4^ regulators have grappled with how best to display this information on packs.^5^ As a result of the former TPD,^6^ packs of cigarettes in the EU were required to display numerical emission yields for tar, nicotine and carbon monoxide. However, emission yields from smoke-machine testing are not valid estimates of human exposure^7^^,^^8^ and some smokers erroneously equate lower tar with reduced harm,^9^ a misperception exploited by tobacco companies.^10^ In the USA, for instance, where the inclusion of machine-produced tar and nicotine yields was optional prior to 2008, between 2000 and 2007 <1% of high tar brands (8 mg or over) displayed tar levels on packs in comparison to more than 85% of low tar brands (3 mg or under).^11^ Consumers have shown a preference for the descriptive display of constituent information about tobacco smoke^5^ and the WHO recommends that Parties ban the display of emission yields on packs and require qualitative statements about emissions, such as the number of carcinogens in smoke.^7^ Following this recommendation, the current TPD prohibits the display of tar, nicotine and carbon monoxide levels on packs and requires a descriptive ‘information’ message explaining that there are more than 70 carcinogens in tobacco smoke.^2^ This applies to all 27 EU countries as well as the UK, which left the EU in January 2020 but had transposed the TPD into the Standardized Packaging of Tobacco Products Regulations^12^ and Tobacco and Related Products Regulations,^13^ which were phased in between 20 May 2016 and 19 May 2017.

**Figure 1:**
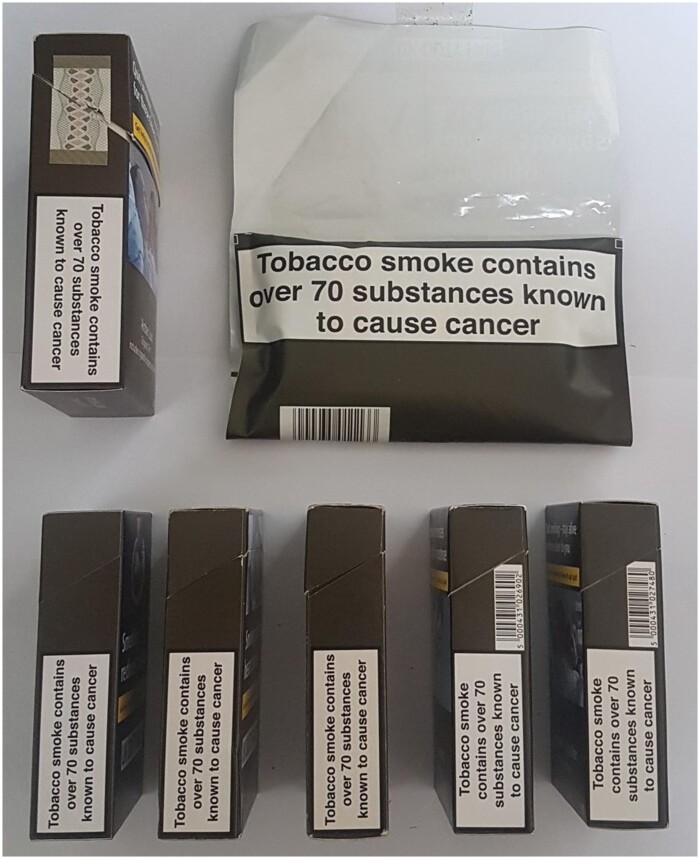
Standardized RYO and cigarette packs

There are differences in how the information message must be displayed on packs of cigarettes and RYO. For cigarettes, the information message is required on one of the lateral surfaces for cuboid packs and shoulder boxes. For RYO, the information message must be displayed on one of the lateral surfaces for cuboid packs or shoulder boxes, the pocket area on the inside of flat-bottomed and wraparound pouches, the base of standing pouches and the inside of the lid for cylindrical packs. In the UK, all cigarettes are sold in cuboid packs, and most RYO in wraparound pouches (see figure 1); in July 2020 most RYO products available in the four leading tobacco-selling supermarkets in the UK were sold in pouches (range 86–92%), with all remaining RYO sold in cuboid packs except for one brand variant (JPS Player’s Real Red) sold in a cylinder. Given that the information message is larger on RYO pouches and packs than on cigarette packs, and as exclusive use of RYO is very common in the UK,^14^ this gave us the opportunity to explore whether exclusive RYO smokers are more likely than exclusive cigarette smokers to know that tobacco smoke contains more than 70 carcinogens.

While scientific understanding of the composition of tobacco smoke, including the number of known carcinogens,^15^ has increased substantially, the same cannot be said for consumer understanding.^16^ A recent systematic review concluded that exploring smokers’ knowledge of constituents in smoke, and whether this increases risk perception and cessation-related behaviours, is a priority.^16^ Since this review a longitudinal online survey with smokers in Australia, Canada and Mexico found that knowledge of toxic constituents in tobacco smoke increased following inclusion of information on warnings on packs, with higher knowledge associated with higher perceived risk of smoking-related conditions.^17^ A randomized controlled study in the USA with smokers who had stickers attached to the side of their cigarette packs providing information about constituents in smoke or about littering (the control group), found that over a 3-week period those with the messages about constituents showed significantly more negative affect, had more conversations about the messages, and were more likely to forgo cigarettes in the past week than those in the control group, but there were no differences in intentions to quit.^18^

We explored any change in smokers’ knowledge of the number of carcinogens in tobacco smoke following the inclusion of an information message on standardized packs in the UK, if this differed between exclusive cigarette and RYO smokers, and whether accurate knowledge of the number of carcinogens in tobacco smoke mediated risk perception, stubbing out cigarettes early, intentions to quit and actual quitting.

## Methods

### Design and sample

The ‘Adult Tobacco Policy Survey’ is a longitudinal online survey, following a cohort of smokers recruited pre-standardized packaging (April–May 2016) and followed up 4–6 months post-standardized packaging (September–November 2017) and 24–26 months post-standardized packaging (May–July 2019). The sample was recruited from the online panel of YouGov, a market research company. To be eligible for inclusion, participants at the first wave had to self-report being a current smoker (either daily, weekly, less than weekly or less than monthly but within the last 3 months). There were 6233 smokers at Wave (W)1 and they were re-contacted at W2 and W3, irrespective of whether they completed W2. A total of 4293 were followed up at W2 (3629 smokers, 607 ex-smokers, 36 non-cigarette smokers, 7 had not smoked in the past 3 months and 14 missing), with 3175 followed up at W3 (2412 smokers, 700 ex-smokers, 44 non-cigarette smokers, 6 had not smoked in the past 3 months and 13 missing). An increased incentive was given at each wave in an attempt to increase retention, with participants receiving 200 points on their YouGov account (equivalent to £2.00) at W1, 300 points at W2 and 400 points at W3. The study received ethical approval from the University of Stirling, with the first two waves approved by the Faculty of Health Sciences and Sport Ethics Committee, and the third wave by the General University Ethical Panel.

### Measures

#### Demographics

Participants reported their age, gender, social grade, household income, highest educational qualification and ethnicity. Age at W1 was recoded into ‘16-24’, ‘25-39’, ‘40-55’ and ‘56 and over’. Social grade was determined by occupation of the main income earner within the household using the National Readership Survey, a classification system in the UK with grades A, B and C1 signifying middle and upper class groups and C2, D and E working class groups.^19^ These grades were recoded into ‘ABC1’ and ‘C2DE’. The 15 categories of ethnicity were recoded into ‘White British’, ‘White non-British’, ‘Other ethnic group’ and ‘Not stated’ for analysis. Household income was categorized as ‘under £30 000’, ‘£30 000 to £44 999’, ‘£45 000 and over’, and ‘Don't know or prefer not to answer’. Highest educational qualification obtained was categorized as ‘High school’, ‘Technical, trade school, A levels, or community college’, ‘University degree or higher degree’ and ‘Don't know or prefer not to say’.

#### Smoking status

At W1 participants were asked about their smoking status to capture whether they smoked cigarettes (factory-made or hand-rolled) every day, smoked cigarettes but not every day, did not smoke cigarettes but used other tobacco products, had never smoked, or had quit. All participants at W1 were cigarette smokers who indicated that they had smoked at least once in the last 3 months.^20^

#### Types of cigarettes consumed

Participants were asked how many cigarettes they usually smoked per day. They were then asked how many of these were rolling tobacco, allowing us to categorize participants as ‘dual users’, ‘exclusive RYO smokers’ or ‘exclusive cigarette smokers’.

#### Heaviness of smoking index (HSI)

Participants were asked ‘On the days that you smoke, how soon after you wake up do you have your first cigarette?’ Within 5 min (coded as 3), 6–30 min (coded as 2), 31–60 min (coded as 1) and after 60 min (coded as 0). The number of cigarettes smoked per day was coded as 10 or fewer (coded as 0), 11–20 (coded as 1), 21–30 (coded as 2) and 31 or more (coded as 3). The two scales were then summed to create a score from 0 to 6.^21^ Missing cases were included as a ‘missing’ category.

#### Awareness of carcinogens

Participants were asked ‘Approximately how many substances do you think there are in tobacco smoke which cause cancer?’ with the response options (None, 1–10, 11–20, 21–30, 31–40, 41–50, 51–60, 61–70, More than 70 and Don’t know) collapsed into ‘70 or fewer’, ‘More than 70’ and ‘Don’t know’.

#### Exaggeration of dangers

Participants were asked ‘To what extent do you agree or disagree with the following statement: The dangers of smoking have been exaggerated?’ with response options (Strongly agree, Agree, Neither agree nor disagree, Disagree, Strongly disagree and Don't know) collapsed into ‘Disagree or strongly disagree’ vs. ‘Other responses including don’t know’.

#### Stubbing out cigarettes

Participants were asked ‘In the last 30 days how many times, if any, did you stub out a cigarette before you finished it because you thought about the harms of smoking?’ with response options (Never, Once, A few times, Many times and Don't know) categorized as ‘Never’ vs. ‘Other responses including don’t know’.

#### Quit intentions

Participants were asked ‘Are you planning to quit smoking?’ with response options (Within the next month, Between 1 and 6 months from now, Sometime in the future, beyond 6 months, Not planning to quit and Don’t know) collapsed into ‘Not planning to quit’ vs. ‘Other responses’.

### Analysis

Frequencies and percentages of participants reporting that smoke contains more than 70 carcinogens were calculated for each wave. Survey weights were provided by YouGov to calibrate the W1 sample to the profile of smokers aged 16 and over in the UK in terms of age, gender and local government office region. Attrition weights were supplied for W2 and W3, accounting for participant loss to follow up. The weighted percentages for each wave are reported by type of product used (RYO, cigarettes or both). The relative change in awareness that there are more than 70 carcinogens in smoke over time in RYO vs. cigarette smokers was assessed using a mixed effects logistic regression with interaction effects between product type and survey wave. For this analysis awareness of carcinogens was recoded into ‘aware that smoke contains more than 70 carcinogens’ vs. ‘other responses including don’t know’. The analysis was adjusted for age group at baseline, gender, heaviness of smoking index (HSI) at baseline, education, household income, occupational group and ethnicity.

Mediation analysis was undertaken to determine whether any changes in disagreement that the dangers of smoking have been exaggerated (risk perception), stubbing out cigarettes early or quit intentions were mediated by a change in awareness that there are more than 70 carcinogens in smoke. Simple comparison of regression coefficients is not possible in logistic regression so the Karlson–Holm–Breen method was employed to account for rescaling in the non-linear models.^22^ The mediation analysis was adjusted for age group at baseline, gender, HSI at baseline, education, household income, occupational group and ethnicity. Analysis of the relationship between quitting and awareness of carcinogens was by logistic regression. The first model had smoking status at W2 as the dependent variable and change in awareness that there are more than 70 carcinogens in smoke between W1 and W2 as the independent variable. The second model had smoking status at W3 as the dependent variable and change in awareness that there are more than 70 carcinogens in smoke between W2 and W3 as the independent variable. The analysis was adjusted for baseline values of the demographic variables previously mentioned. Where proportions are presented these are weighted proportions but measures of association, such as odds ratios, are unweighted. Analyses were undertaken in Stata version 15.

## Results

### Sample characteristics

The characteristics of the samples at each wave are shown in detail in Supplementary table S1.

### Awareness of the number of carcinogens in tobacco smoke by product type

Among dual smokers (those smoking both cigarettes and RYO), the weighted proportion who indicated that tobacco smoke contains more than 70 carcinogens increased at each wave (28.0% at W1, 33.9% at W2 and 39.6% at W3), see table 1. An increase across waves was also found for exclusive cigarette smokers (23.7% at W1, 33.9% at W2 and 39.7% at W3) and exclusive RYO smokers (27.5% at W1, 36.5% at W2 and 45.1% at W3), with the increase from W1 to W3 significantly greater among exclusive RYO smokers than exclusive cigarette smokers [adjusted odds ratio (AOR) =1.44, 95% CI 1.03–2.03].

**Table 1:** Awareness of the number of carcinogens in tobacco smoke among cigarette, RYO and dual smokers

	**Number of carcinogens** **in tobacco smoke**	**Wave 1** ^a^	**Wave 2** ^a^	**Wave 3** ^a^
Dual smokers	70 or fewer	469 (51.8%)	205 (46.1%)	125 (47.0%)
	More than 70	237 (28.0%)	151 (33.9%)	102 (39.6%)
	Don’t know	194 (20.2%)	95 (20.0%)	45 (13.4%)
	Total	900 (100%)	451 (100%)	272 (100%)
Exclusive cigarette smokers	70 or fewer	1527 (49.0%)	751 (39.4%)	452 (34.5%)
	More than 70	721 (23.7%)	602 (33.9%)	453 (39.7%)
	Don’t know	894 (27.3%)	531 (26.7%)	342 (25.8%)
	Total	3142 (100%)	1884 (100%)	1247 (100%)
Exclusive RYO smokers	70 or fewer	930 (45.2%)	451 (39.0%)	248 (31.4%)
	More than 70	532 (27.5%)	432 (36.5%)	355 (45.1%)
	Don’t know	584 (27.3%)	316 (24.6%)	211 (23.5%)
	Total	2046 (100%)	1199 (100%)	814 (100%)

a Number excluded for responding ‘Don’t know’ to the question asking how many of the cigarettes they smoked were RYO cigarettes: 145 (2.3%) W1, 95 (2.6%) W2, 79 (3.3%) W3.

### Awareness of the number of carcinogens in tobacco smoke and risk perception, stubbing out cigarettes and intention to quit

In a model, adjusted for age group, gender, HSI, education, household income, occupational group and ethnicity, the odds of disagreeing that the dangers of smoking had been exaggerated (risk perception) were 23% greater in W3 relative to W1 (see table 2). When the awareness of the number of carcinogens in tobacco smoke variable was included in the model the difference between W1 and W3 was no longer significant (AOR = 1.07, 95% CI 0.91–1.27), suggesting that knowing that tobacco smoke contains more than 70 carcinogens mediates the change in risk perception. Mediation analysis confirmed this, indicating that 65.7% of the difference in risk perception between W1 and W3 was mediated through change in knowledge that tobacco smoke contains more than 70 carcinogens.

**Table 2:** Mixed effects model of disagreement that the risks of smoking have been exaggerated by survey wave, adjusted for gender, heaviness of smoking, age group, household income, education, ethnic group and occupational group

Variable	Value	Odds Ratio	95% CI	
			Lower	Upper
Wave	Wave 1 (ref)	1		
	Wave 2	0.85	0.74	0.98
	Wave 3	1.23	1.04	1.46
Gender	Female (ref)	1		
	Male	1.75	1.40	2.18
Heaviness of	0	1		
Smoking	1	0.34	0.23	0.50
Index	2	0.29	0.20	0.40
	3	0.31	0.22	0.43
	4	0.25	0.16	0.37
	5	0.22	0.12	0.40
	6	0.11	0.04	0.31
	Missing	0.09	0.04	0.23
Age	16–24 (ref)	1.00		
	25–39	1.45	0.94	2.21
	40–55	0.98	0.64	1.51
	56 and older	0.46	0.29	0.71
Household income	Under £30 000 (ref)	1.00		
	£30 000–£44 999	1.38	1.08	1.76
	£45 000 and over	1.36	1.01	1.83
	Don't know/prefer not to say	0.65	0.50	0.84
Educational qualification	High school or less (ref)	1.00		
	Technical, trade school, A levels, community college	2.00	1.55	2.59
	At least university degree	2.96	2.30	3.81
	Don't know/prefer not to say	0.76	0.48	1.22
Ethnic group	White British (ref)	1.00		
	White other	0.69	0.42	1.13
	Other ethnic group	0.39	0.23	0.65
	Prefer not to say	0.18	0.06	0.55
Occupational social group	ABC1 (ref)	1.00		
	C2DE	0.65	0.53	0.80
	Refused or unknown	1.15	0.70	1.89
	Constant	2.20	1.23	3.95

a Number of individuals in GEE analysis =6233, number of observations =12 274.

Not stubbing out cigarettes early was less likely in W2 than W1 (AOR = 0.84, 95% CI 0.73–0.96) but not significantly different in W3 than in W1 (AOR = 0.86, 95% CI 0.74–1.01), see Supplementary table S2. The odds of having an intention to quit did not change across waves (W1 vs. W3 AOR = 1.13, 95% CI 0.96–1.33), see Supplementary table S3. Mediation analysis showed that the change in stubbing out early was not mediated by knowledge that tobacco smoke contains more than 70 carcinogens. The relationship between awareness that there are more than 70 carcinogens in smoke and stubbing out early is shown in table 3. Although the weighted proportion of participants who were aware that there are more than 70 carcinogens in smoke increased across waves in all groups, it increased by about the same amount in people who stubbed cigarettes out early and those who did not. For those who stubbed out early the increase in awareness that smoke contains more than 70 carcinogens between W1 and W3 was 13.1% (24.8–37.9%), while for those who did not stub cigarettes out early the increase was 16.0% (25.7–41.7%), see table 3. This was tested in a fully adjusted mediation analysis and there was no statistically significant mediation. There was no change in quit intentions across waves so a mediation analysis was not performed. The weighted proportions of those who intended to quit who were aware that there are more than 70 carcinogens in smoke are shown in table 3.

**Table 3:** Risk perception, stubbing out early and quit intentions by survey wave and awareness of carcinogens

	W1 total *n*	W1 *n* weighted % aware there are >70 carcinogens	W2 total *n*	W2 *n* weighted % aware there are >70 carcinogens	**W3 total** ***n***	W3 *n* weighted % aware there are >70 carcinogens
Dangers not exaggerated	3742	1083 (30.0%)	2048	806 (40.5%)	1424	648 (48.4%)
Dangers exaggerated/don’t know	2491	429 (18.3%)	1581	395 (25.7%)	988	277 (28.5%)
Has stubbed out early/don’t know	1839	452 (24.8%)	1105	362 (32.4%)	705	270 (37.9%)
Has not stubbed out early	4394	1060 (25.7%)	2523	838 (35.0%)	1707	655 (41.7%)
Intention to quit/don’t know	4538	1188 (27.4%)	2552	883 (34.8%)	1658	673 (42.2%)
No intention to quit	1695	324 (19.9%)	1076	317 (32.6%)	754	252 (37.1%)
Total	6233	1512	3628	1200	2412	925

### Awareness of the number of carcinogens in tobacco smoke and quitting

Among ex-cigarette smokers, the proportion aware that there are more than 70 carcinogens in smoke was 37.5% at W2 and 40.5% at W3, similar to dual smokers (33.9% at W2 and 39.6% at W3), see table 1. The logistic regression analysis did not indicate that change in awareness of there being more than 70 carcinogens in smoke was associated with quitting.

## Discussion

We found that accurate knowledge of the number of carcinogens in tobacco smoke increased among smokers in the UK following the inclusion of an information message on standardized packs, and mediated higher perception of risk but not stubbing out cigarettes early, intentions to quit or quitting.

While warnings on the side of cigarette packs, such as those currently used in the USA, are less salient and effective than warnings on the front and back of packs,^23,^^24^ our findings show that the inclusion of health messaging on the side of cigarette packs can increase awareness of this information. That exclusive RYO smokers were significantly more likely than exclusive cigarette smokers to correctly identify tobacco smoke as containing more than 70 carcinogens also points to the value of using the inside of pouches to communicate with smokers. While we are not able to determine whether RYO smokers used cuboid packs or pouches, RYO is typically sold in pouches in the UK. With sales of RYO growing in European countries not subject to the TPD, as well as in most other regions (Asia, Africa, Australia, Middle East and Central America),^25^ the findings suggest that in addition to using the main display areas of packs, countries should also require the inside flap of pouches to be used to communicate health messages. Irrespective of product or pack type that the constituent message appeared on standardized packs may have increased salience given that it does not have to compete with full branding.

Consistent with a longitudinal survey in Australia, Canada and Mexico, not only did knowledge of constituents in smoke increase following inclusion of this information on packs, but greater knowledge was associated with higher perceived risk.^17^ We found that knowing that smoke contains more than 70 carcinogens mediated change in risk perception, indicating that 65.7% of the difference in risk perception between W1 and W3 was mediated through increased knowledge of the number of carcinogens in tobacco smoke. Similarly, just as a 3-weeks randomized controlled trial found that smokers exposed to messages about constituents were not more likely to intend to quit than those exposed to a control message,^18^ we found that micro-indicators of behaviour change (stubbing out cigarettes early and intention to quit) and actual behaviour change (quitting) were not mediated by accurate knowledge of carcinogens in tobacco smoke. In Australia and New Zealand, seven rotating messages are required on the side of cigarette packs, they must be in black text on a yellow background to increase salience,^26^ and cover the total area of one side of the pack, except for the area covering the flip-top,^27^^,^^28^ see Supplementary figure S1. Research exploring whether this approach has an impact on cessation-related behaviours would be of significant value.

While surveys taken from online panels are increasingly commonly in academic research, and recent (past 3 months) internet use in the UK is over 90%,^29^ as internet access is lowest among the most deprived this group may be less likely to be part of online panels. The data are based on self-report. With longitudinal surveys, respondent fatigue is a potential limitation.^30^ Attrition is also a problem with longitudinal research, which reduces the precision of survey estimates,^30^^,^^31^ with approximately half (49%) the original sample lost by W3.

A systematic review suggested that well-presented constituent information on packs could increase knowledge and perceptions of risk, and potentially change behaviour.^16^ We found that the inclusion of an information message explaining that tobacco smoke contains more than 70 carcinogens on packs increased knowledge and perceptions of risk, providing some support for the WHO recommendation to use this type of additional messaging on packs, but it did not change behaviour. As only a single text message about constituents in smoke is required on cigarette and RYO packs, the UK Government and European Commission could build upon this by following the approach taken by Australia and New Zealand and requiring a series of more prominent messages.

## Supplementary data

Supplementary data are available at *EURPUB* online.

## Funding 

The first two waves were funded by Cancer Research UK and the British Heart Foundation (Grant No: A18507). The third wave was conducted by the Public Health Policy Research Unit (PH-PRU), commissioned and funded by the National Institute for Health Research Policy Research Programme.

## Disclaimers 

The views expressed are those of the authors and not necessarily those of the NHS, the National Institute for Health Research, the Department of Health and Social Care or its arm's length bodies, and other Government Departments.

*Conflicts of interest*: None declared.

**Key points**

- Packs of cigarettes and rolling tobacco in the UK are required to display an ‘information message’ explaining the number of carcinogens in tobacco smoke.
- Accurate knowledge of the number of carcinogens in tobacco smoke increased among smokers in the UK following the inclusion of this information on standardized packs, with the greatest increase for exclusive roll-your-own smokers.
- As rolling tobacco becomes more popular globally governments should require the inside of pouches to be used to communicate health messaging.
- Knowledge of the correct number of carcinogens in tobacco smoke mediated risk perceptions, but not stubbing out cigarettes, intending to quit or actual quitting.

## Supplementary Material

ckab101_Supplementary_Data
