# Supplementary material for: The impacts of including information about the number of carcinogens in smoke on standardized cigarette packs in the UK
Source: Eur J Public Health. 2021 Sep 14;31(5):1031–7. doi: 10.1093/eurpub/ckab101 (PMC8546877; doi:10.1093/eurpub/ckab101)
Supplement: ckab101_Supplementary_Data [file ckab101_supplementary_data.zip › ejph-2020-08-om-1006-File004.docx]

**Supplementary Table 1:** Characteristics of the sample by survey wave

|  |  | **All** | | | **Cigarette smokers** | | |
| --- | --- | --- | --- | --- | --- | --- | --- |
|  |  | **Wave** | | | **Wave** | | |
| **Education** | | **1** | **2** | **3** | **1** | **2** | **3** |
|  | High school or less | 2032 | 1465 | 1096 | 2032 | 1271 | 859 |
|  | % | 32.6 | 34.1 | 34.5 | 32.6 | 35.0 | 35.6 |
|  | Technical, trade school, A levels, community college | 1610 | 991 | 720 | 1610 | 837 | 535 |
|  | % | 25.8 | 23.1 | 22.7 | 25.8 | 23.2 | 22.2 |
|  | At least university degree | 2396 | 1693 | 1270 | 2396 | 1407 | 948 |
|  | % | 38.4 | 39.4 | 40.0 | 38.4 | 38.8 | 39.3 |
|  | Don't know or prefer not to say | 195 | 144 | 89 | 195 | 114 | 70 |
|  | % | 3.1 | 3.4 | 2.8 | 3.1 | 3.1 | 2.9 |
| **Gender** |  |  |  |  |  |  |  |
|  | Male | 2889 | 2006 | 1519 | 2889 | 1687 | 1153 |
|  | % | 46.4 | 46.7 | 47.8 | 46.4 | 46.5 | 47.8 |
|  | Female | 3344 | 2287 | 1656 | 3344 | 1942 | 1259 |
|  | % | 53.7 | 53.3 | 52.2 | 53.7 | 53.5 | 52.2 |
| **Gross household income** | |  |  |  |  |  |  |
|  | Under £30,000 | 2840 | 1909 | 1359 | 2840 | 1657 | 1058 |
|  | % | 45.6 | 44.5 | 42.8 | 45.6 | 45.7 | 43.9 |
|  | £30,000 to £44,999 | 1294 | 894 | 664 | 1294 | 743 | 506 |
|  | % | 20.8 | 20.8 | 20.9 | 20.8 | 20.5 | 21.0 |
|  | £45,000 and over | 969 | 665 | 563 | 969 | 549 | 407 |
|  | % | 15.6 | 15.5 | 17.7 | 15.6 | 15.1 | 16.9 |
|  | Don't know or prefer not to answer | 1130 | 825 | 589 | 1130 | 680 | 441 |
|  | % | 18.1 | 19.2 | 18.6 | 18.1 | 18.7 | 18.3 |
| **Ethnic group** | |  |  |  |  |  |  |
|  | White British | 5499 | 3842 | 2863 | 5499 | 3255 | 2175 |
|  | % | 88.2 | 89.5 | 90.2 | 88.2 | 89.7 | 90.2 |
|  | White other | 352 | 210 | 141 | 352 | 172 | 104 |
|  | % | 5.7 | 4.9 | 4.4 | 5.7 | 4.7 | 4.3 |
|  | Other | 318 | 196 | 137 | 318 | 163 | 105 |
|  | % | 5.1 | 4.6 | 4.3 | 5.1 | 4.5 | 4.4 |
|  | Prefer not to say | 64 | 45 | 34 | 64 | 39 | 28 |
|  | % | 1.0 | 1.1 | 1.1 | 1.0 | 1.1 | 1.2 |
| **Age group** | |  |  |  |  |  |  |
|  | 16 to 24 | 650 | 181 | 82 | 650 | 132 | 57 |
|  | % | 10.4 | 4.2 | 2.6 | 10.4 | 3.6 | 2.4 |
|  | 25 to 39 | 1795 | 1089 | 682 | 1795 | 867 | 486 |
|  | % | 28.8 | 25.4 | 21.5 | 28.8 | 23.9 | 20.2 |
|  | 40 to 55 | 2053 | 1497 | 1140 | 2053 | 1287 | 876 |
|  | % | 32.9 | 34.9 | 35.9 | 32.9 | 35.5 | 36.3 |
|  | 56 and over | 1735 | 1497 | 1271 | 1735 | 1343 | 992 |
|  | % | 27.8 | 34.9 | 40.0 | 27.8 | 37.0 | 41.1 |
| **Social grade** | |  |  |  |  |  |  |
|  | ABC1 | 3583 | 2407 | 1815 | 3583 | 2034 | 1363 |
|  | % | 57.5 | 56.1 | 57.2 | 57.5 | 56.1 | 56.5 |
|  | C2DE | 2472 | 1771 | 1314 | 2472 | 1497 | 1019 |
|  | % | 39.7 | 41.3 | 41.4 | 40.0 | 41.3 | 42.3 |
|  | Refused or unknown | 178 | 115 | 46 | 178 | 98 | 30 |
|  | % | 2.9 | 2.7 | 1.5 | 2.9 | 2.7 | 1.2 |
| **Aware that smoke contains 70 carcinogens** | | | |  |  |  |  |
|  | No | 2992 | 1726 | 1132 | 2992 | 1455 | 859 |
|  | % | 48.0 | 40.2 | 35.7 | 48.0 | 40.1 | 35.6 |
|  | Yes | 1512 | 1443 | 1219 | 1512 | 1201 | 925 |
|  | % | 24.3 | 33.6 | 38.4 | 24.3 | 33.1 | 38.4 |
|  | Don’t know | 1729 | 1124 | 824 | 1729 | 973 | 628 |
|  | % | 27.7 | 26.2 | 26.0 | 27.7 | 26.8 | 26.0 |
| **Dangers of smoking exaggerated** | | |  |  |  |  |  |
|  | Strongly agree | 162 | 117 | 70 | 162 | 102 | 55 |
|  | % | 2.6 | 2.7 | 2.2 | 2.6 | 2.8 | 2.3 |
|  | Agree | 701 | 463 | 301 | 701 | 414 | 247 |
|  | % | 11.3 | 10.8 | 9.5 | 11.3 | 11.4 | 10.2 |
|  | Neither agree nor disagree | 1404 | 1042 | 707 | 1404 | 935 | 596 |
|  | % | 22.5 | 24.3 | 22.3 | 22.5 | 25.8 | 24.7 |
|  | Disagree | 2279 | 1449 | 1106 | 2279 | 1223 | 823 |
|  | % | 36.6 | 33.8 | 34.8 | 36.6 | 33.7 | 34.1 |
|  | Strongly disagree | 1463 | 1068 | 884 | 1463 | 825 | 601 |
|  | % | 23.5 | 24.9 | 27.8 | 23.5 | 22.7 | 24.9 |
|  | Don’t know | 224 | 154 | 107 | 224 | 130 | 90 |
|  | % | 3.6 | 3.6 | 3.4 | 3.6 | 3.6 | 3.7 |
| **Stubbing out early** | |  |  |  |  |  |  |
|  | Have stubbed out early or don’t know | 1839 | 1105 | 706 | 1839 | 1105 | 705 |
|  | % | 29.5 | 30.5 | 29.3 | 29.5 | 30.5 | 29.2 |
|  | Have never stubbed out early | 4394 | 2523 | 1708 | 4394 | 2523 | 1707 |
|  | % | 70.5 | 69.5 | 70.8 | 70.5 | 69.5 | 70.7 |
| **Intention to quit** | |  |  |  |  |  |  |
|  | Intention to quit or don’t know | 4538 | 2552 | 1659 | 4538 | 2552 | 1658 |
|  | % | 72.8 | 70.3 | 68.7 | 72.8 | 70.3 | 68.7 |
|  | No intention to quit | 1695 | 1076 | 755 | 1695 | 1076 | 754 |
|  | % | 27.2 | 29.7 | 31.3 | 27.2 | 29.7 | 31.3 |

**Supplementary Table 2:** Mixed effects model of stubbing cigarettes out early by survey wave

| Variable | Value | Odds Ratio | 95% CI | |
| --- | --- | --- | --- | --- |
|  |  |  | **Lower** | **Upper** |
| Wave | Wave 1 (ref) | 1.00 |  |  |
|  | Wave 2 | 0.84 | 0.73 | 0.96 |
|  | Wave 3 | 0.86 | 0.74 | 1.01 |
| Gender | Female (ref) | 1.00 |  |  |
|  | Male | 0.66 | 0.55 | 0.79 |
| Heaviness of | 0 | 1.00 |  |  |
| Smoking | 1 | 0.88 | 0.65 | 1.20 |
| Index | 2 | 0.64 | 0.49 | 0.84 |
|  | 3 | 1.43 | 1.10 | 1.85 |
|  | 4 | 2.18 | 1.55 | 3.06 |
|  | 5 | 3.87 | 2.24 | 6.70 |
|  | 6 | 7.98 | 2.94 | 21.62 |
|  | Missing | 0.42 | 0.20 | 0.88 |
| Age | 16 to 24 (ref) | 1.00 |  |  |
|  | 25 to 39 | 0.87 | 0.61 | 1.22 |
|  | 40 to 55 | 1.04 | 0.74 | 1.47 |
|  | 56 and older | 0.76 | 0.54 | 1.09 |
| Household income | Under £30,000 (ref) | 1.00 |  |  |
|  | £30,001 to £44,999 | 0.93 | 0.76 | 1.15 |
|  | £45,000 and over | 0.92 | 0.72 | 1.19 |
|  | Don't know/prefer not to say | 0.90 | 0.72 | 1.13 |
| Educational qualification | High school or less (ref) | 1.00 |  |  |
|  | Technical, trade school, A levels, community college | 0.98 | 0.79 | 1.23 |
|  | At least university degree | 0.99 | 0.80 | 1.23 |
|  | Don't know/prefer not to say | 0.70 | 0.47 | 1.06 |
| Ethnic group | White British (ref) | 1.00 |  |  |
|  | White other | 0.97 | 0.65 | 1.44 |
|  | Other ethnic group | 0.49 | 0.33 | 0.74 |
|  | Prefer not to say | 0.63 | 0.26 | 1.53 |
| Occupational social group | ABC1 (ref) | 1.00 |  |  |
|  | C2DE | 1.09 | 0.91 | 1.30 |
|  | Refused or unknown | 0.67 | 0.44 | 1.04 |
|  | Constant | 11.15 | 6.81 | 18.28 |

**Supplementary Table 3:** Mixed effects model of intention to quit smoking by survey wave

| Variable | Value | Odds Ratio | 95% CI | |
| --- | --- | --- | --- | --- |
|  |  |  | **Lower** | **Upper** |
| Wave | Wave 1 (ref) | 1.00 |  |  |
|  | Wave 2 | 1.07 | 0.93 | 1.24 |
|  | Wave 3 | 1.13 | 0.96 | 1.33 |
| Gender | Female (ref) | 1.00 |  |  |
|  | Male | 0.49 | 0.40 | 0.61 |
| Heaviness of | 0 | 1.00 |  |  |
| Smoking | 1 | 0.33 | 0.23 | 0.48 |
| Index | 2 | 0.30 | 0.22 | 0.43 |
|  | 3 | 0.49 | 0.36 | 0.66 |
|  | 4 | 1.04 | 0.71 | 1.53 |
|  | 5 | 1.65 | 0.92 | 2.96 |
|  | 6 | 2.96 | 1.15 | 7.58 |
|  | Missing | 1.65 | 0.69 | 3.92 |
| Age | 16 to 24 (ref) | 1.00 |  |  |
|  | 25 to 39 | 0.47 | 0.31 | 0.71 |
|  | 40 to 55 | 1.10 | 0.73 | 1.65 |
|  | 56 and older | 1.96 | 1.29 | 2.98 |
| Household income | Under £30,000 (ref) | 1.00 |  |  |
|  | £30,001 to £44,999 | 0.99 | 0.78 | 1.25 |
|  | £45,000 and over | 0.74 | 0.55 | 1.00 |
|  | Don't know/prefer not to say | 0.94 | 0.73 | 1.22 |
| Educational qualification | High school or less (ref) | 1.00 |  |  |
|  | Technical, trade school, A levels, community college | 0.75 | 0.58 | 0.96 |
|  | At least university degree | 0.62 | 0.48 | 0.79 |
|  | Don't know/prefer not to say | 0.73 | 0.46 | 1.17 |
| Ethnic group | White British (ref) | 1.00 |  |  |
|  | White other | 1.68 | 1.05 | 2.68 |
|  | Other ethnic group | 1.00 | 0.60 | 1.64 |
|  | Prefer not to say | 3.78 | 1.35 | 10.55 |
| Occupational social group | ABC1 (ref) | 1.00 |  |  |
|  | C2DE | 1.23 | 1.01 | 1.50 |
|  | Refused or unknown | 0.94 | 0.57 | 1.54 |
|  | Constant | 0.62 | 0.35 | 1.09 |
