# Supplementary figures and images for: The impacts of including information about the number of carcinogens in smoke on standardized cigarette packs in the UK
Source: Eur J Public Health. 2021 Sep 14;31(5):1031–7. doi: 10.1093/eurpub/ckab101 (PMC8546877; doi:10.1093/eurpub/ckab101)

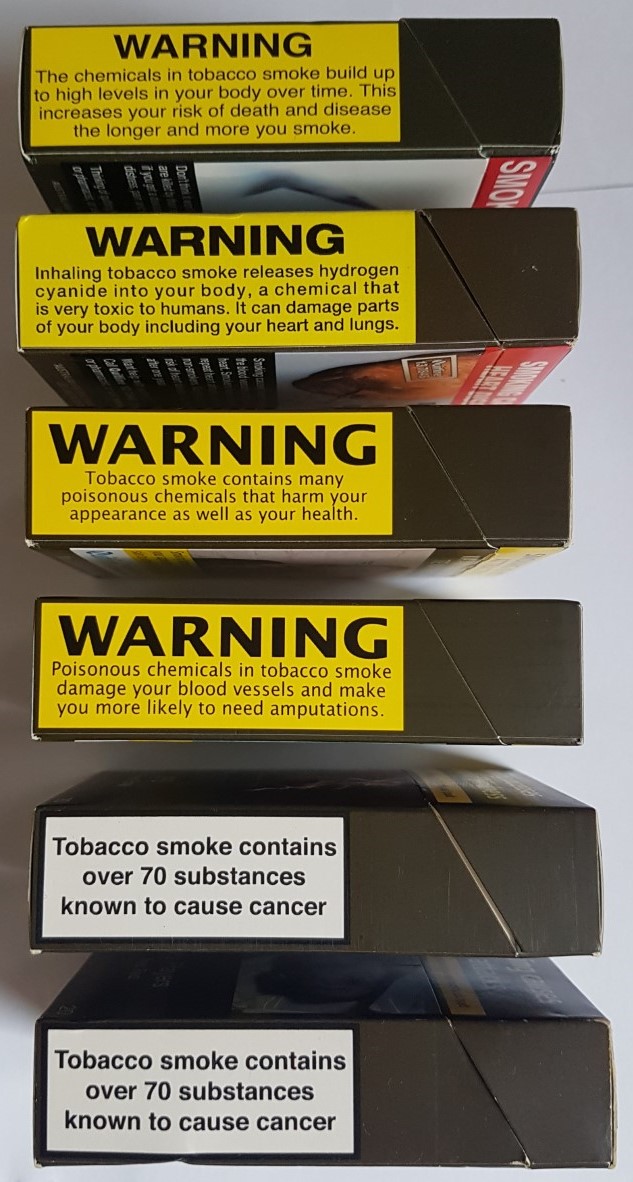

Supplement: ckab101_Supplementary_Data [file ckab101_supplementary_data.zip › ejph-2020-08-om-1006-File003.jpg]
